# Efficacy of the combination of MEK and CDK4/6 inhibitors *in vitro* and *in vivo* in KRAS mutant colorectal cancer models

**DOI:** 10.18632/oncotarget.9153

**Published:** 2016-05-04

**Authors:** Michael S. Lee, Timothy L. Helms, Ningping Feng, Jason Gay, Qing Edward Chang, Feng Tian, Ji Y. Wu, Carlo Toniatti, Timothy P. Heffernan, Garth Powis, Lawrence N. Kwong, Scott Kopetz

**Affiliations:** ^1^ Division of Hematology and Oncology, Department of Medicine, University of North Carolina at Chapel Hill, Chapel Hill, NC, USA; ^2^ Lineberger Comprehensive Cancer Center, Chapel Hill, NC, USA; ^3^ Department of Genomic Medicine, University of Texas MD Anderson Cancer Center, Houston, TX, USA; ^4^ Department of Gastrointestinal Medical Oncology, University of Texas MD Anderson Cancer Center, Houston, TX, USA; ^5^ Department of Applied Cancer Science, University of Texas MD Anderson Cancer Center, Houston, TX, USA; ^6^ Center for Co-Clinical Trials, University of Texas MD Anderson Cancer Center, Houston, TX, USA; ^7^ Sanford-Burnham Medical Research Institute, La Jolla, CA, USA

**Keywords:** KRAS, NRAS, MEK inhibitor, CDK4/6 inhibitor

## Abstract

**Purpose:**

Though the efficacy of MEK inhibitors is being investigated in *KRAS*-mutant colorectal cancers (CRC), early clinical trials of MEK inhibitor monotherapy did not reveal significant antitumor activity. Resistance to MEK inhibitor monotherapy developed through a variety of mechanisms converging in ERK reactivation. Since ERK increases cyclin D expression and increases entry into the cell cycle, we hypothesized that the combination of MEK inhibitors and CDK4/6 inhibitors would have synergistic antitumor activity and cause tumor regression *in vivo*.

**Results:**

The combination of MEK and CDK4/6 inhibitors synergistically inhibited cancer cell growth *in vitro* and caused tumor regression *in vivo* in cell line and patient-derived xenograft models. Combination therapy markedly decreased levels of phosphorylated ribosomal protein S6 both *in vitro* and *in vivo* and decreased Ki67 staining *in vivo*.

**Experimental Design:**

We performed *in vitro* proliferation, colony formation, apoptosis, and senescence assays, and Western blots, on a panel of 11 *KRAS* mutant CRC cell lines treated with the MEK inhibitor MEK162, the CDK4/6 inhibitor palbociclib, or the combination. We also treated 4 *KRAS* mutant CRC cell line and patient-derived xenografts with the MEK inhibitor trametinib, the CDK4/6 inhibitor palbociclib, or the combination, and performed immunohistochemical and reverse phase protein array analysis.

**Conclusions:**

Combined inhibition of both MEK and CDK4/6 is effective in preclinical models of *KRAS* mutant CRC and justifies a planned phase II clinical trial in patients with refractory *KRAS*-mutant CRC.

Efficacy of the combination of MEK and CDK4/6 inhibitors *in vitro* and *in vivo* in *KRAS* mutant colorectal cancer models.

## INTRODUCTION

Somatic activating mutations in *KRAS* or *NRAS* are present in up to 52% of colorectal cancer (CRC) [1, 2], causing constitutive activation of the RAF/MEK/ERK signaling pathway independent of upstream receptor tyrosine kinases like the epidermal growth factor receptor (EGFR). These mutations are known predictive biomarkers of resistance in metastatic CRC to anti-EGFR therapy, such as cetuximab or panitumumab, and consequently patients whose tumors harbor *KRAS* or *NRAS* mutations have fewer therapeutic options. There are currently no known effective therapies that exploit *KRAS* or *NRAS* mutations to target malignant cells.

Though MEK inhibitors were found to have intriguing *in vitro* activity in *KRAS* mutated CRC models [3–6], efficacy was variable in different cell lines. More importantly, MEK inhibitor monotherapy proved to be largely ineffective in patient-derived xenograft (PDX) murine models [7] and in human clinical trials [8]. Myriad mechanisms of resistance have been identified [9–13], with most causing increased signaling through upstream receptor tyrosine kinases or activation of parallel signal transduction cascades to bypass or overcome MEK inhibition and reactivate ERK signaling. Indeed, unless an approximately ten-fold reduction in ERK activation is achieved, cell proliferation persists [14]. Consequently, rational drug combinations with MEK inhibitors, potentially with agents that target downstream effectors of ERK, are likely necessary to overcome compensatory responses to MEK inhibitors.

Phosphorylation and activation of ERK is well-known to cause increased proliferation and increased activity of the cell cycle by causing increased cyclin D expression [15, 16]. Cyclin D expression is the rate-limiting step in cell cycle progression from G1 into S phase [17]. Cyclin D complexes with and activates cyclin dependent kinase (CDK) 4 and 6, which phosphorylate and inactivate the tumor suppressor retinoblastoma protein (Rb) [18]. In its unphosphorylated state, Rb is bound to the transcription factor E2F, and Rb phosphorylation releases E2F, freeing it to increase transcription of genes promoting cell cycle progression into S phase. Selective CDK4/6 inhibitors have been developed with minimal off-target kinase inhibition [19–21], with the caveat that they require intact expression of Rb for antitumor efficacy [19]. However, inactivating mutations in *RB1* are very uncommon in CRC and do not exceed the expected background rate of mutations [1], and in fact the majority of CRCs have higher levels of Rb than normal colon mucosa [22]. Furthermore, loss of Rb speeds cell growth in conjunction with RAS mutations [23–25], indicating that RAS mutant cell growth increases with unimpeded, dysregulated cell cycle progression. Thus, RAS mutant CRCs are a candidate for further investigation of the efficacy of CDK4/6 inhibitors. The combination of CDK4/6 and MEK inhibitors may be particularly efficacious in RAS mutated malignancies. Inhibition of CDK4 was found to be synthetically lethal *in vitro* and *in vivo* in *KRAS* mutant non-small cell lung cancers [26, 27]. However, CDK4/6 inhibitor monotherapy in early human clinical trials did not yield any responses in CRC [28–31], arguing that combination therapy is required. In an inducible *NRAS* Q61K genetically engineered mouse model of melanoma, the combination of MEK and CDK4/6 inhibitors caused tumor regression that paralleled extinction of mutant *NRAS*, but either monotherapy alone did not [32]. Consequently, we hypothesized that the combination of a CDK4/6 inhibitor and a MEK inhibitor would have synergistic antitumor activity *in vitro* and *in vivo* in *KRAS* mutant CRC.

## RESULTS

### Dual inhibition of MEK and CDK4/6 markedly attenuates cell growth *in vitro*

**Figure 1:**
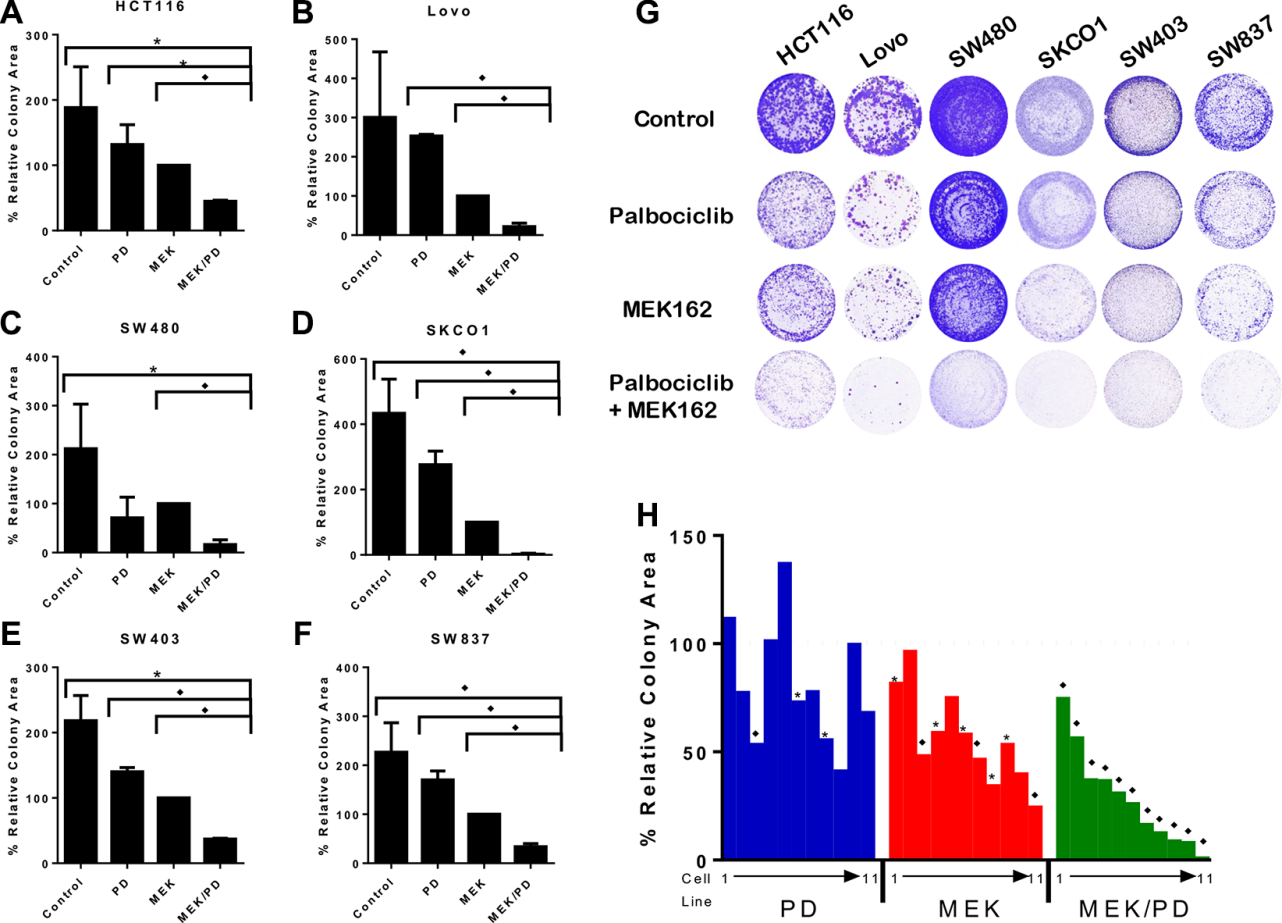
Combination of MEK and CDK4/6 inhibitors markedly attenuates cell growth *in vitro* in a panel of *KRAS* mutant CRC cell lines (**A**–**F**), Colony assays for six representative cell lines treated with the CDK4/6 inhibitor palbociclib (PD), the MEK inhibitor MEK162 (MEK), the combination (MEK/PD), or with DMSO control for 10–14 days. Results are normalized to MEK162 monotherapy. The data represent mean values ± SD for 3–4 independent experiments. (**G**), Depiction of the stained cell wells. (**H**), Summary colony assay results for a panel of 11 *KRAS* mutant CRC cell lines treated with DMSO control, PD, MEK, or MEK/PD at concentrations approximating the IC_50_ for MEK monotherapy for 10–14 days. Results here are normalized to DMSO control. A waterfall plot is depicted showing values, with cell lines consistently depicted in the same order of T84, LS174T, SW1116, SW948, LS1034, HCT116, SW837, SW403, SW480, Lovo, and SKCO1. The data represent mean values for 3–4 independent experiments. *p < 0.05, *p < 0.006. All *p*-values were generated by Student's *t*-test.

To examine the efficacy of dual blockade of CDK4/6 and MEK on *KRAS* mutant CRC cells, cell growth and colony formation were determined after treatment with the MEK inhibitor MEK162 and the CDK4/6 inhibitor palbociclib, using clinically relevant doses of palbociclib [28, 29] and doses optimized to each cell line to maximally display contrast of cell growth between monotherapies and combination therapy (See Supplementary Table S1 and Figure S1). As shown in Figure 1A–1G, the combination of MEK162 and palbociclib was markedly effective in attenuating cell growth and colony formation in a broad panel of *KRAS* mutant CRC cell lines. As shown in Figure 1H, the combination of MEK inhibitor and CDK4/6 inhibitor was more effective in limiting colony formation and cell growth than MEK inhibitor monotherapy in the majority of the 11 *KRAS* mutant CRC cell lines assayed.

### Dual inhibition of MEK and CDK4/6 synergistically inhibits growth of *KRAS* mutant colon cancer cell lines

**Figure 2:**
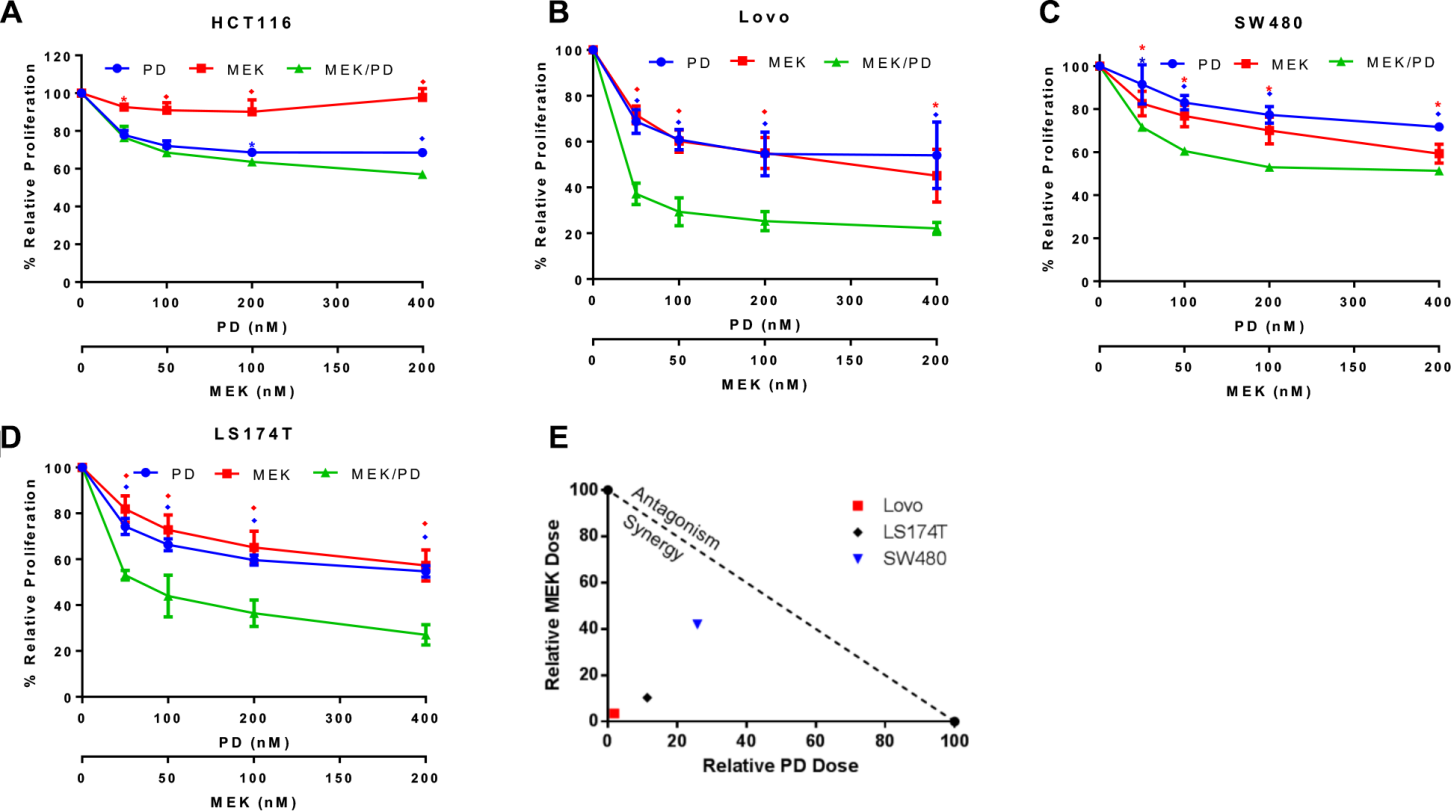
Combination of MEK and CDK4/6 inhibitors synergistically attenuates cell growth in a panel of *KRAS* mutant CRC cell lines (**A**), HCT116 cells were treated with the CDK4/6 inhibitor palbociclib (PD) (400 nM, 200 nM, 100 nM, or 50 nM) and/or the MEK inhibitor MEK162 (MEK) (200 nM, 100 nM, 50 nM, or 25 nM respectively) or control for 72 hours and proliferation and cell growth was assessed using MTS assay. The data represent mean values ± SD for 3 independent experiments. (**B**), Lovo cells treated as described for HCT116. (**C**), SW480 cells treated as described for HCT116. (**D**), LS174T cells treated as described for HCT116. (**E**), Isobologram shows synergy of the combination of PD0332991 and MEK162. Plotted points farther to the bottom left of the Figure represent increasing degree of synergy. *p < 0.05, ♦*p* < 0.006. All *p*-values were generated by Student's *t*-test.

To examine the mechanism of dual blockade of CDK4/6 and MEK on *KRAS* mutant CRC cells, cell growth was assessed with MTS assay after treatment with MEK162 and palbociclib for 72 hours. The cell lines HCT116, Lovo, SW480, and LS174T showed improved efficacy of the combination of MEK162 and palbociclib compared to either monotherapy, with the effect reaching significance for Lovo, SW480, and LS174T at all concentration levels tested (Figure 2A–2D). On assessment for formal synergy, these three cell lines all displayed synergistic effects (combination index, CI, at EC_50_ for Lovo was 0.05, for SW480 was 0.68, and for LS174T was 0.22; CI values < 1 indicate synergy). An isobologram for these findings is depicted in Figure 2E.

**Figure 3:**
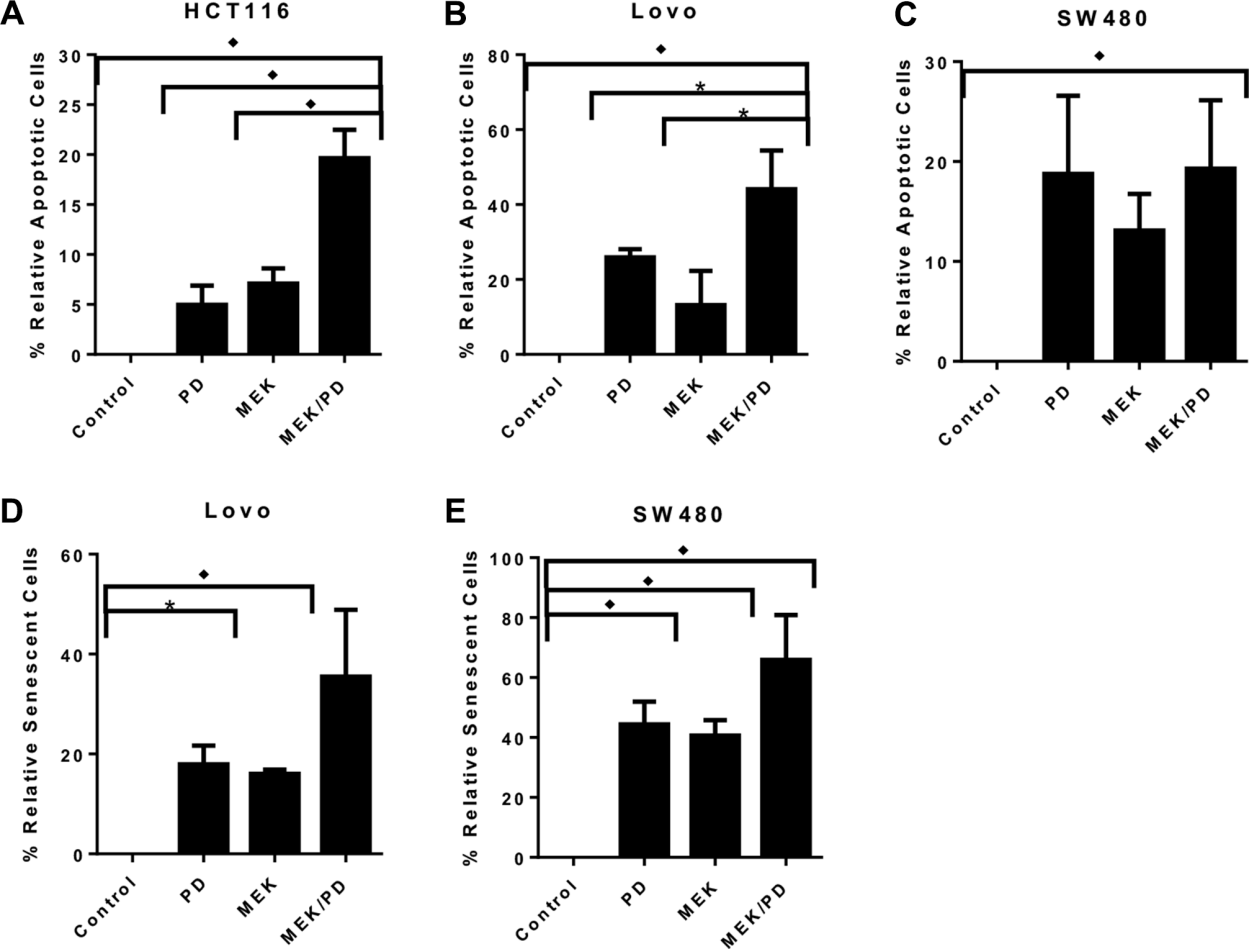
Combination of MEK and CDK4/6 inhibitors inhibits cell growth in *KRAS* mutant CRC cell lines through a variety of mechanisms (**A**), HCT116 cells were treated with the CDK4/6 inhibitor palbociclib (PD, 400 nM), the MEK inhibitor MEK162 (MEK, 200 nM), the combination (MEK/PD), or with DMSO control for 72 hours, and stained with propidium iodide and annexin V-FITC prior to performing flow cytometry. The data represent mean values ± SD for 3 independent experiments. (**B**), Lovo cells were treated as described for HCT116. C, SW480 cells were treated as described for HCT116. *p < 0.05. ♦*p* < 0.01 for (**A**–**C**), with all *p*-values generated by Student's *t*-test. (**D**), Lovo cells were treated with PD (400 nM), MEK (200 nM), the combination, or with DMSO control for 72 hours, and stained with X-gal to detect senescence-associated β-galactosidase. The proportion of stained cells in 10 random high-powered fields was counted. The data shows mean values from a representative experiment ± SD for the 10 fields. (**E**), SW480 cells were treated as described for Lovo. ♦*p* ≤ 0.0002 for combination compared to any other value, with all *p*-values generated by Student's *t*-test.

Using a flow-based annexin V-FITC assay, treatment with the combination of CDK4/6 inhibitor and MEK inhibitor for 72 hours induced apoptosis in a greater proportion of cells than either monotherapy in HCT116 and Lovo cell lines, but not in SW480 and SW403 (Figure 3A–3B; SW403 not shown). The combination also induced senescence in a greater proportion of cells after 72 hours as determined by staining for senescence-associated beta-galactosidase, for Lovo and SW480 (Figure 3D–3E), while there was negligible staining in HCT116 cells regardless of treatment (not shown), and SW403 could not be assessed because of cell clumping precluding accurate counting. Overall, these results suggest that the synergy of the drug combination acts through some combination of increased apoptosis and/or senescence, at least *in vitro*.

### Dual inhibition of MEK and CDK4/6 results in greater inhibition of phosphorylation of ribosomal protein S6

**Figure 4:**
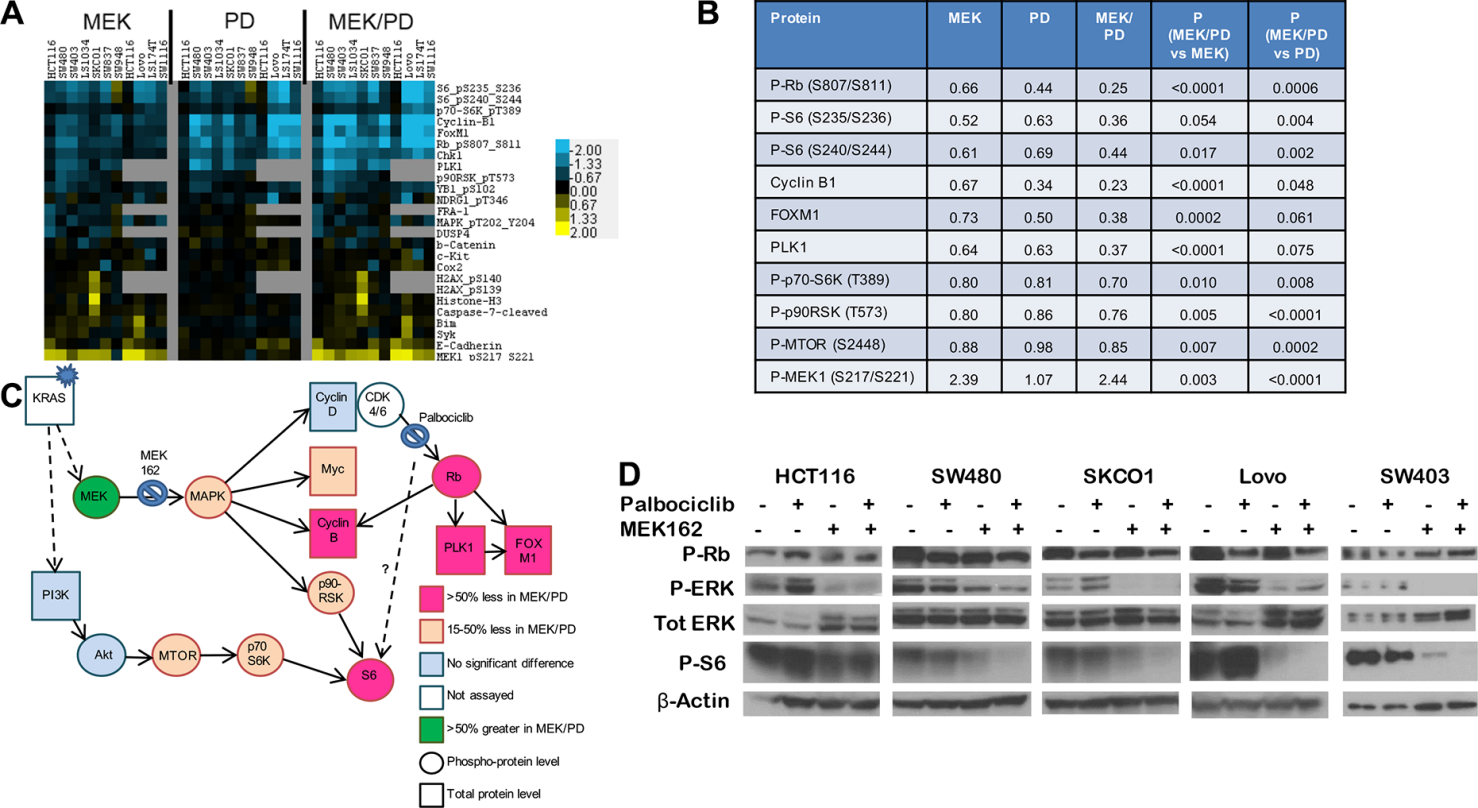
Combination of CDK4/6 and MEK inhibitors induces greater inhibition of phosphorylation of S6 and other growth factor signaling and cell cycle proteins (**A**), Protein lysates were obtained from a panel of 9 *KRAS* mutant CRC cell lines after treatment with DMSO control, palbociclib (PD) 400 nM, MEK162 (MEK) 200 nM, or MEK162 200 nM + palbociclib 400 nM (MEK/PD) for 24 hours, and RPPA was performed. Protein levels were normalized to DMSO controls of each cell line, and a log2 heatmap of the most differentially expressed proteins was generated. (**B**), Median expression levels of proteins in *KRAS* mutant CRC cell lines treated with MEK162 (MEK) alone, palbociclib (PD) alone, or the combination (MEK/PD), relative to DMSO-treated control. *p*-values were determined using paired Student's *t*-test. (**C**), Protein expression data from RPPA was mapped according to relevant pathways. Circles represent phosphorylated epitopes on the proteins and squares represent total proteins. Activating *KRAS* mutations are thought to lead to constitutive activation of downstream signaling pathways, but adding MEK inhibitor and CDK4/6 inhibitor yielded the depicted changes in protein expression. (**D**), Western blotting of the indicated antibodies for *KRAS* mutant cell lines HCT116, SW480, SKCO1, Lovo, and SW403 treated with palbociclib 400 nM and/or MEK162 200 nM for 24 hours.

To determine mechanisms of action and inform on pharmacodynamic markers of response, we performed reverse phase protein arrays (RPPA) using protein lysates obtained from 10 *KRAS* mutant CRC cell lines treated for 24 hours with MEK162, palbociclib, the combination, or DMSO control (Figure 4A–4B). Protein changes after MEK inhibitor treatment were in line with previous publications using RPPA [32, 33], including downregulation of Myc and Fra1, upregulation of Bim, and a feedback increase in MEK phosphorylation.

Consistent with the selectivity of palbociclib for CDK4/6, only five proteins were consistently affected by palbociclib across all lines, including significant downregulation of the proliferation markers Rb, Foxm1, Plk1, and Ccnb1 (Cyclin B1). Palbociclib was consistently better than or at least as efficient as MEK162 in suppressing these markers. Importantly, the drug combination more strongly downregulated these markers than either monotherapy alone in every cell line tested, Figure 4B suggesting a cooperative antiproliferative effect.

Unexpectedly, palbociclib alone also resulted in the inhibition of phosphorylation of ribosomal protein S6 in 8 of the 10 lines, which has not been reported previously. MEK162 alone also downregulated pS6, consistent with known signaling, and the effect was more marked with the drug combination in all cell lines except HCT116. These findings were recapitulated by immunoblotting for 5 of the lines (Figure 4D) including HCT116. In SW480, SKCO1, Lovo, and SW403, pS6 was nearly or completely undetectable after combination treatment. Notably, pS6 has been identified as a marker of *in vivo* responsiveness to MEK inhibitors in BRAF-mutant melanoma [34]. Thus, the muted combination effect in HCT116 on pS6 is consistent with the observed lack of formal drug synergy (Figure 2A). Furthermore, the combination demonstrated slight but significant cooperative downregulation in components upstream of pS6, including phospho-mTOR, phospho-p70-S6 kinase, and p90 ribosomal S6 kinase (p90^rsk^) (see Figure 4B, 4C). These findings together suggest a degree of cross-talk or feedback between CDK4/6 signaling and mTOR.

As S6 also lies downstream of the PI3K pathway, we next asked whether activation of PI3K by PIK3CA mutations affected the pS6 response to combination therapy. We noted that of three PIK3CA mutant cell lines, HCT116 and SW948 had relatively less pS6 downregulation than PIK3CA wild-type cell lines, but that the third, LS174T, had strong pS6 downregulation (see Figure S2). Consequently, though *PIK3CA* mutation may be associated with shallower changes to phospho-S6 on treatment, it may not be completely sufficient or fully penetrant in blocking pS6 downregulation.

### Dual inhibition of MEK and CDK4/6 inhibits tumor growth *in vivo* in *KRAS* mutant CRC cell line xenograft and PDX

**Figure 5:**
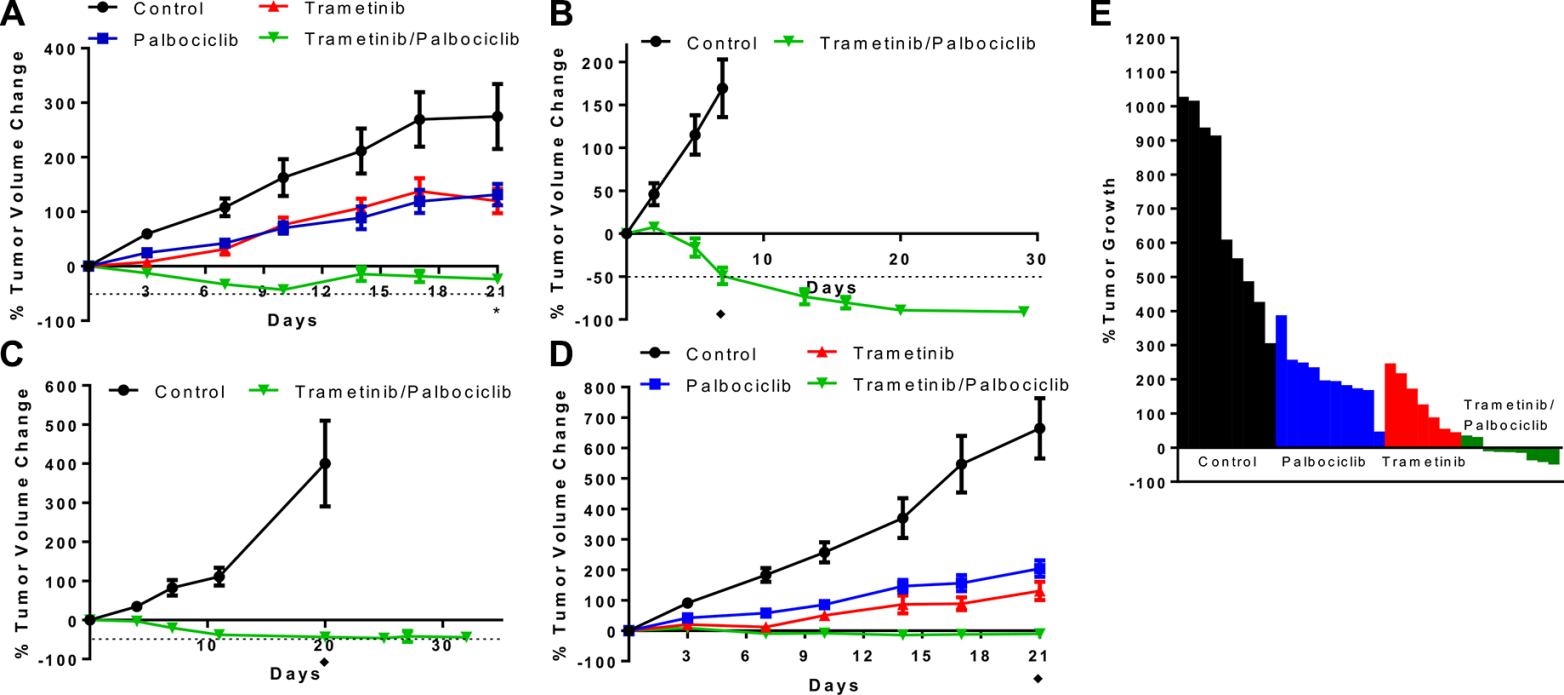
Combination of CDK4/6 and MEK inhibitors induces tumor regression *in vivo* in *KRAS* mutant CRC xenografts (**A**), Lovo cell line xenografts were treated with vehicle control daily, palbociclib 100 mg/kg daily, trametinib 3 mg/kg every 2 days, or palbociclib 100 mg/kg daily + trametinib 3 mg/kg every 2 days, and tumor volume was measured twice per week. Data are mean ± SEM, with 10 mice/arm. *p < 0.01 on day 21 for combination vs. each other arm by Student's *t*-test. (**B**), HCT116 cell line xenografts were treated with control chow or chow containing trametinib + palbociclib, and tumor volume was measured longitudinally. ♦*p* < 0.001 on day 7 by Student's *t*-test. (**C**), SW480 cell line xenografts were treated with control chow or chow containing trametinib + palbociclib, and tumor volume was measured longitudinally. ♦*p* < 0.001 on day 20 by Student's *t*-test. (**D**), Xenografts implanted with patient-derived CRC cells harboring *KRAS* A146T mutation were treated with vehicle control daily, palbociclib 100 mg/kg daily, trametinib 3 mg/kg every 2 days, or palbociclib 100 mg/kg daily + trametinib 3 mg/kg every 2 days, and tumor volume was measured twice per week. Data are mean ± SEM, with 9–10 mice/arm. ♦*p* < 0.001 on day 21 for combination vs. any other arm by Student's *t*-test. Dotted lines represent 50% decrease in tumor volume. (**E**), Change in tumor volume for each of the patient-derived xenograft mice in D at day 21 of treatment.

We next tested the combination *in vivo* using CRC xenograft models. The combination of the MEK and CDK4/6 inhibitors trametinib (3 mg/kg QOD) and palbociclib (100 mg/kg QD) using oral gavage yielded significantly greater tumor growth inhibition in murine xenografts of the *KRAS* mutant Lovo cell line compared to vehicle-treated controls or treatment with monotherapy of each drug alone (see Figure 5A). The combination treatment did cause weight loss in mice, with a maximum weight loss tending to occur at days 10 through 14. Importantly though, mice with less weight lost showed weight stabilization and/or complete recovery with continued treatment (see Figure S3A), suggesting that alternative dosing schedules may improve toxicity. We also developed a trametinib/palbociclib chow calculated to deliver a similar dose to oral gavage continuously. Lovo xenografts again showed regression upon treatment with this combination chow, demonstrating similar efficacy to oral gavage (see Figure S3B). Independent cell line xenografts of HCT116 and SW480 also showed tumor regression in mice treated with the combination chow (see Figure 5B–5C). No tumors showed resistance to the combination at 30+ days of treatment. Furthermore, these mice did not demonstrate weight loss exceeding 20% or morbidity with the combination chow despite demonstrating tumor shrinkage.

We also leveraged a *KRAS* mutant CRC PDX model that we have recently established. Similar to the results in the Lovo cell line xenograft model, the combination of MEK and CDK4/6 inhibitors yielded significantly greater tumor growth inhibition in the PDX model compared to vehicle-treated controls or treatment with monotherapy of each drug alone (see Figure 5D). Notably, this PDX model responded to the combination despite harboring an atypical *KRAS* A146T mutation and an activating AKT1 E17K mutation. The combination treatment yielded regression of tumors after 21 days, unlike treatment with either monotherapy. The combination treatment again caused weight loss in mice, with a nadir weight between days 7 and 14 before stabilization and partial recovery of the lost weight (see Figure S3C).

**Figure 6:**
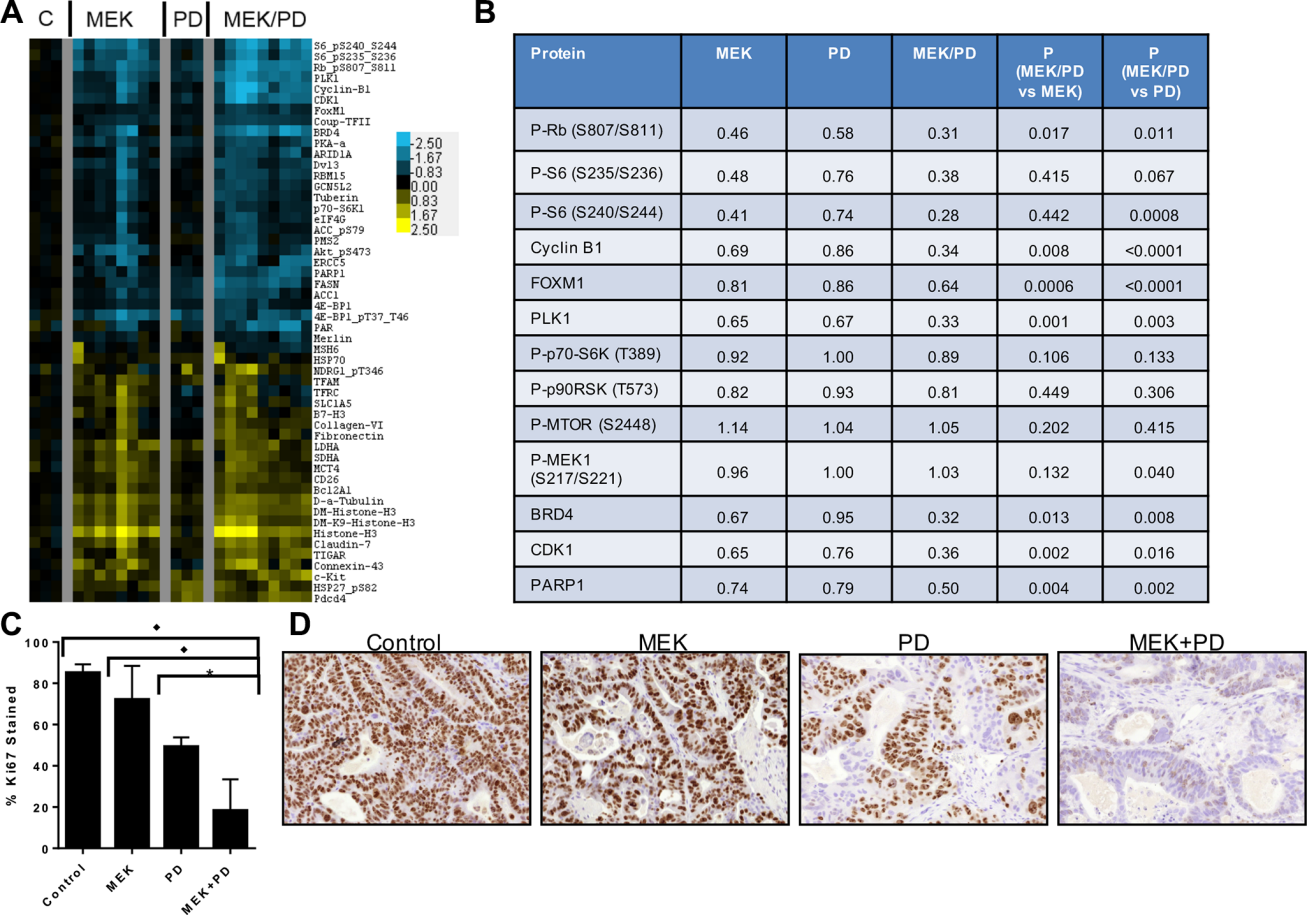
Combination of CDK4/6 and MEK inhibitors induces greater inhibition of phosphorylation of S6 and other growth factor signaling and cell cycle proteins *in vivo* in PDX models of *KRAS* mutant CRC (**A**), Protein lysates were obtained from tumors after 21 days of treatment with vehicle control (C), trametinib (MEK), palbociclib (PD), or trametinib + palbociclib (MEK/PD), and RPPA was performed. Protein levels were normalized to the geometric mean of vehicle-treated controls, and a heatmap of the most differentially expressed proteins was generated. Values are log2-transformed fold-change compared to the geometric mean of the controls. (**B**), Median expression levels of selected proteins in *KRAS* mutant CRC PDXs treated with trametinib (MEK) alone, palbociclib (PD) alone, or the combination (MEK/PD) for 21 days, relative to vehicle-treated control. *p*-values were determined using paired Student's *t*-test. (**C**), Percentage of nuclear area stained by Ki67 antibody. Data represent mean ± SD of samples from 3–4 mice. *p ≤ 0.02, ♦*p* < 0.003 by Student's *t*-test. (**D**), Representative immunohistochemistry images for Ki67 staining.

To molecularly compare the *in vivo* inhibition with our earlier *in vitro* studies, RPPA was performed using protein lysates obtained from PDX tumors after treatment for 21 days (Figure 6A–6B). Similar to the *in vitro* data, treatment with the combination of MEK and CDK4/6 inhibitors downregulated Rb, Plk1, Ccnb1, and pS6 to a greater extent than either monotherapy alone. Interestingly, unlike in the *in vitro* model, treatment with trametinib did not show a feedback increase in phosphorylation of MEK (see Figure 6B) after 21 days of treatment. Additionally, a number of proteins showed cooperative changes upon combination therapy that were not observed *in vitro* including CDK1 and FASN, suggesting the influence of the tumor microenvironment on inhibitor-induced protein changes. Immunohistochemical staining from PDX samples demonstrated a marked decrease in cell staining for Ki67 after 21 days of treatment (see Figure 6C–6D). Staining for phospho-S6 was decreased in the trametinib and the combination trametinib + palbociclib treated groups compared to control in this chronically-treated cohort (see Figure S4). Collectively, these data support the hypothesis that the combination of MEK and CDK4/6 inhibitors induces tumor regression via the cooperative induction of cell cycle arrest, and that Ki67 and pS6 may serve as surrogates for combined target engagement.

## DISCUSSION

Though mutations in *KRAS* and other RAS isoforms are widespread in many malignancies, as yet efforts to therapeutically target *KRAS* mutations have been unsuccessful. Previous studies investigating the use of MEK inhibitor monotherapy revealed that though resistance may arise through a variety of mechanisms, generally they result in compensatory activation of parallel signaling pathways to reactivate phosphorylation of ERK [9–13]. ERK plays a key role in promoting cell cycle progression and proliferation [35], including through increased transcription of cyclin D1 [15, 16]. Several synthetic lethality screens have been performed on *KRAS* mutant cancer cell lines to determine potential targets, and results have frequently included genes important in cell cycle progression or mitosis, such as CDK1 [36], cyclin A2 [37], PLK1 [37], and CDC6 [38]. Furthermore, abrogation of CDK4 was synthetically lethal in *KRAS* mutant non-small cell lung cancer models [26, 27]. Consequently, we hypothesized that the combination of inhibition of the MAPK pathway with a MEK inhibitor and inhibition of cell cycle progression with a CDK4/6 inhibitor would be more effective. Indeed, our studies demonstrate that combined inhibition of MEK and CKD4/6 is synergistic *in vitro* in a variety of *KRAS* mutant CRC cell lines and yields tumor regression *in vivo* in cell line xenografts and PDXs of *KRAS* mutant CRC.

It was previously not well recognized that CDK4/6 inhibition would inhibit phosphorylation of S6, which is more typically associated with growth factor signaling through the PI3K/MTOR and MEK/ERK pathways. Our results have most notably found that both inhibition of MEK and CDK4/6 individually cause decreases in phospho-S6 levels, and the combination yields even greater decrease. Ribosomal protein S6 is a component of the 40S ribosomal subunit that can be phosphorylated at five serine residues by S6 kinase in the mTOR pathway or by p90^rsk^ in an ERK-dependent manner [39–41]. Phosphorylated S6 is associated with increased cell size and increased translation of polypeptides encoded by mRNAs with a 5′-polypyrimidine tract [41]. Entry into the cell cycle is associated with increased phosphorylation of S6 at Ser240/244 [42], and so decrease in phospho-S6 may reflect generally decreased flux through the cell cycle. Nevertheless, decreased phospho-S6 level upon combination treatment appears to be a pharmacodynamic biomarker of therapy that warrants further evaluation.

Mechanisms of action of CDK4/6 inhibitors also remain under investigation. Decreases in the levels of cyclin B1, FOXM1, and PLK1 were observed with the combination of CDK4/6 inhibitors and MEK inhibitors. Similar changes in levels of downstream target genes have been found *in vitro* in estrogen receptor-positive breast cancer cell lines treated with palbociclib combined with the aromatase inhibitor letrozole [43]. Decreases of these effectors are important in mediating cellular fate. CDK4 and CDK6 phosphorylate and stabilize the transcription factor FOXM1 to maintain G1/S phase gene expression and suppress senescence [44], so decreased levels of FOXM1 likely contribute toward shifting the cell into senescence. PLK1 is a kinase important in promoting mitotic entry and proliferation [45], and its transcription is upregulated by E2F [46], and thus decreased levels of PLK1 are expected with decreased phospho-RB. Cyclin B1 complexes with CDK1 to promote mitotic entry, and accumulation of cyclin B1 depends on E2F target genes [47], so decrease in phospho-RB levels would also likely result in decreased cyclin B1 levels.

A previous study of palbociclib in *KRAS* mutant pancreatic cancer cell lines showed concern that though palbociclib monotherapy inhibited cell proliferation, it appeared to also increase epithelial mesenchymal transition (EMT) in cell lines with wild-type *SMAD4* [48]. In that study, CDK4/6 inhibition or knockdown resulted in induction of the EMT markers beta-catenin, Slug, N-cadherin, and vimentin. However, in our experiments with both *KRAS* mutant CRC cell lines and *KRAS* mutant PDX, there were no significant changes in protein levels of E-cadherin, N-cadherin, or beta-catenin *in vitro* or *in vivo*. Notably, *in vivo* results were obtained after 21 days of treatment. Several of the cell lines used, including HCT116 and Lovo, were wild-type in *SMAD4* [49], as was the PDX tested. *In vitro*, there were also no differences in vimentin or fibronectin. There was a trend toward increased fibronectin level *in vivo* with combination MEK and CDK4/6 inhibitors that was not observed with CDK4/6 inhibitor monotherapy. This result may be different than the *in vitro* result due to the longer duration of treatment in the *in vivo* experiment or due to stromal cells increasing fibronectin expression. While increased fibronectin is commonly associated with EMT, fibronectin levels may also be altered due to other phenomena, and increased fibronectin expression has even been associated with increased cell senescence [50], so elevated fibronectin in isolation is not sufficient to state there is an increase in EMT. Indeed, inhibition of CDK4 in a triple-negative breast cancer cell line caused decreases in the EMT-associated transcription factors Snail and Twist and decreases in phosphorylated Smad3 [51, 52]. Thus, differences in EMT or other feedback pathways in response to CDK4/6 inhibition are likely to be cell-type-specific, but warrant further investigation.

Though the combination of CDK4/6 inhibitors and MEK inhibitors has previously been found to be effective in *NRAS*-mutant melanoma models [32], we felt that empirically assessing the efficacy of the combination in *KRAS*-mutant CRC models was necessary to justify consideration of clinical trials in patients with CRC, especially given the many distinctions between RAS-mutated melanomas and CRCs. First, RAS-mutated melanoma is predominantly *NRAS*-mutated at codon 61, while RAS-mutated CRC is predominantly *KRAS*-mutated at codons 12 and 13. *NRAS* and *KRAS* mutations have distinct phenotypic effects on resulting tumors in CRC genetically engineered mouse models [53], and mutations at different codons even within *NRAS* cause different phenotypes [54]. Second, RAS-mutant melanoma and CRC have markedly different patterns of concomitant genomic alterations, such as *CDKN2A* loss [55, 56], which are likely to be associated with sensitivity to CDK4/6 inhibitors [57]. Third, MEK inhibitor monotherapy is more effective in clinical trials of *NRAS*-mutant melanoma than in *KRAS*-mutant CRC, as early phase III trial data of MEK162 (binimetinib) in *NRAS*-mutant melanoma showed the study met its primary endpoint of improvement in progression-free survival (unpublished data), while MEK inhibitors had no evidence of efficacy in phase I studies in RAS-mutant CRCs [58]. Finally, the tissue-specific context of oncogene mutations is clearly critical in determining sensitivity to targeted therapies; notably, single-agent BRAF inhibitors or combination of BRAF and MEK inhibitors are effective in*BRAF*-mutant melanomas [59, 60], while they are not effective in *BRAF*-mutant CRCs [61, 62]. Given these concerns, our data demonstrated preclinical efficacy of combination MEK and CDK4/6 inhibitors in RAS-mutant CRCs. This data also complements the recent report of the combination of palbociclib and trametinib in *KRAS*-mutant CRC PDX models [63], but demonstrates that inhibition of phospho-S6 is a potential pharmacodynamic biomarker, and uniquely demonstrates that this inhibition is synergistic with the combination of CDK4/6 and MEK inhibitors in a broader number and mutational diversity of cell lines, including “atypical” *KRAS* A146T mutant CRC PDX models. Our data has justified development of a planned phase II clinical trial of the combination in patients with refractory metastatic RAS-mutant CRC.

In summary, we evaluated a novel combination of MEK and CDK4/6 inhibitors in *KRAS* mutant CRCs and found synergistic inhibition of cell growth *in vitro*, with contributions of decreased proliferation, increased apoptosis, and increased senescence; and we subsequently found the combination of MEK and CDK4/6 inhibitors caused tumor regression *in vivo* in cell line and patient-derived xenografts. We also described inhibition of phosphorylation of ribosomal protein S6 to a greater extent with the combination of MEK and CDK4/6 inhibitors compared to either alone. Though our studies were focused on *KRAS* mutant CRC, this does not rule out activity in RAS wild-type or *BRAF* mutant CRC, and further study is warranted. Given the more limited number of options in treatment of *KRAS* mutant CRC compared to *KRAS* wild-type CRC, the development of effective therapies for this patient population is an area of unmet need. Combinatorial approaches targeting both the MAPK pathway and cell cycle regulatory pathways merit further preclinical and clinical investigation.

## MATERIALS AND METHODS

### Cell culture and drugs

A panel of *KRAS* mutant CRC cell lines was cultured in 1:1 DMEM/Ham's F12 media with 10% fetal bovine serum, 1% penicillin/streptomycin, and 2 mM L-glutamine. Palbociclib/PD0332991 (Selleck Chemicals) and MEK162 (Selleck Chemicals) were dissolved in dimethyl sulfoxide (DMSO) as 10 mM and 30 mM stocks, respectively. Lovo cells were obtained from ATCC. HCT116, SW480, SKCO1, SW403, SW837, LS174T, SW948, SW1116, LS1034, and T84 cell identity was verified by short tandem repeat (STR) analysis.

### Cell proliferation and colony formation assays

Cells were seeded in a 96-well plate at 2000–6000 cells/well, incubated overnight at 37°C to allow adhesion, and then treated with inhibitors for 72 hours. Cell proliferation was determined using MTS solution (Promega), and formal assessment for synergy performed per the Chou-Talalay method [64, 65] using CompuSyn (ComboSyn, Inc, Paramus, NJ). For colony formation assay, cells were seeded in a 6-well plate at 8000–80,000 cells/well, incubated overnight at 37°C to allow adhesion, and then treated with inhibitors for 2–3 weeks. Cell colonies were fixed with ice-cold methanol and stained with 1% crystal violet. The density of colonies over the plate area was quantified by ImageJ (NIH) [66]. For detection of exposed phosphatidylserine residues reflective of apoptosis, Annexin V-FITC apoptosis assay kit (BD Biosciences) was used. Cells were seeded at 500,000 cells/well in 6-well plates, incubated overnight at 37°C to allow adhesion, and then treated with inhibitors for 72 hours. Cells were washed and stained with annexin V-FITC and propidium iodide, and flow cytometry was performed using the Gallios flow cytometer (Beckman Coulter) and analyzed with Kaluza flow analysis software (Beckman Coulter). To measure cell senescence, treated cells were stained for senescence-associated beta-galactosidase (Chemicon). Cells were seeded at 500,000 cells/well in 6-well plates, incubated overnight at 37°C to allow adhesion, and then treated with inhibitors for 72 hours. Cells were washed with PBS, fixed with glutaraldehyde and methanol, washed twice more, and then incubated overnight in the dark at 37°C under ambient atmospheric conditions with X-Gal. Subsequently, cells were washed, and 10 high-powered light microscopy images of each well were captured, and cells with blue staining were manually counted.

### Protein analysis

Reverse-phase protein array analysis (RPPA) was performed as previously described [67]. Briefly, cells were seeded at 500,000 cells/well in 6-well plates, incubated overnight at 37°C to allow adhesion, and then treated with inhibitors for 24 hours. Cellular proteins were collected and denatured by 1% sodium dodecyl sulfate (SDS) with β-mercaptoethanol. The ratio of values for each treatment within each cell line compared to control was determined. To construct a heatmap, these ratios were log2 transformed, and proteins with at least two cell lines with at least 1.74-fold increase or decrease of protein expression compared to control were filtered. Protein values were clustered using Gene Cluster 3.0 [68], and heatmap was created using Java Treeview version 1.1.6r4 (http://jtreeview.sourceforge.net) [69].

For immunoblotting, phospho-p42/44 ERK T202/Y204, p42/44 ERK1/2, phospho-RB Ser780, RB, and phospho-S6 Ser235/236 were purchased from Cell Signaling Technology (Danvers, MA). Anti-beta-actin was obtained from Sigma-Aldrich. Anti-mouse and anti-rabbit horseradish peroxidase-linked secondary antibodies were obtained from Cell Signaling Technology.

### *In vivo* murine xenografts

CD-1 nude 6-week old female mice (Charles River) were injected with 0.2 ml Lovo cell suspension subcutaneously in the right flank and monitored for tumor growth. After tumors were established with median tumor volume exceeding 100 mm^3^, treatment of 10 mice/arm was commenced via oral gavage with either vehicle control per os daily, trametinib 3 mg/kg per os every 2 days, palbociclib 100 mg/kg per os daily, or combination of trametinib 3 mg/kg per os every 2 days and palbociclib 100 m/kg per os daily for 21 days. Tumor size and mouse weight were measured every 3–4 days. After 21 days, treatment was discontinued and mice were sacrificed.

Additionally, immunodeficient female nude mice were injected with one million cells of HCT116 or SW480 subcutaneously in the flank and monitored for tumor growth. After tumors were established with median tumor volume exceeding 100 mm^3^, treatment of 5–8 mice/arm was commenced with chow containing combination trametinib and palbociclib or control chow. The chow was designed to deliver a dose equivalent to 0.75 mg/kg trametinib and 75 mg/kg palbociclib. Tumor size and mouse weight were measured every 3–5 days. Control mice were sacrificed after 11 days due to tumor enlargement causing morbidity. After 29–32 days, treatment was discontinued and mice were sacrificed.

### Patient-derived xenografts

Primary human-tumor xenograft models were established as previously described [70]. Tumor specimens were obtained from patients with metastatic colorectal cancer at the University of Texas MD Anderson Cancer Center, and all patients provided informed written consent for specimens to be used for research purposes including implantation in xenografts. Samples were obtained with approval of the institutional review board. Xenografts were propagated in NU/J 6-week old female mice (Jackson Laboratory). After tumors were established with median tumor volume exceeding 300 mm^3^, treatment of 10 mice/arm was commenced via oral gavage with either vehicle control daily, trametinib 3 mg/kg every 2 days, palbociclib 100 mg/kg daily, or combination of trametinib 3 mg/kg every 2 days and palbociclib 100 m/kg daily for 21 days. Tumor size and mouse weight were measured every 3–4 days. After 21 days, treatment was discontinued and mice were sacrificed. Tumors from 3–4 mice per arm were excised, segmented, and immediately flash frozen in liquid nitrogen. Frozen tumor tissue was homogenized in lysis buffer (1% Triton X-100, 50 mM HEPES at pH 7.4, 150 mM NaCl, 1.5 mM MgCl_2_, 1 mM EGTA, 100 mM NaF, 10 mM sodium pyrophosphate, 1 mM Na_3_VO_4_, 10% glycerol, protease and phosphatase inhibitors) and centrifuged. Protein concentration was determined, and lysates were denatured with 4 × SDS sample buffer with 10% beta-mercaptoethanol, and a final volume of 50 μl of 1 μg/μl denature protein lysate was sent for RPPA as described above.

### Immunohistochemical stains

Five-micron thick sections were cut and mounted from a formalin fixed paraffin-embedded tissue block on microscope slides and baked at 60°C for 1 hour. Slides were deparaffinized in xylene 3 times for 5 minutes each, washed with 100% ethanol 3 times for 5 minutes each, and rehydrated in a series of 95%, 70%, 50% ethanol dilutions in distilled water for 5 minutes each. Slides were heated in 10 mM sodium citrate, pH 6.0, with 0.05% Tween-20 in a Decloaker chamber (Biocare Medical, Concord, CA) at 95°C for 30 minutes, then cooled to 90°C for 10 seconds. For each stain, slides were washed with 3% H_2_O_2_ in 1 × TBS for 10 minutes, then in Rodent Block M (Biocare Medical) for 20 min, and then with primary antibody for 30 minutes at room temperature. Primary antibodies were rabbit anti-Ki67 (Thermo Scientific) at 1:500 dilution or rabbit anti-phospho S6 ribosomal protein (Ser235/236) (Cell Signaling) at 1:200 dilution. Afterward, slides were incubated with Rabbit-on-Rodent HRP-polymer (Biocare Medical) for 25 minutes, stained with DAB for 5 minutes, and washed with deionized water. Slides were counterstained with Harris hematoxylin for 60 seconds and washed under water for 1 minute, and then dehydrated in 2 changes of 95% ethanol, 3 changes of 100% ethanol, and 3 changes of xylene. Images of representative areas of the slides were captured. The percentage of nuclear area stained for Ki67 was quantitated using the ImmunoRatio plugin for ImageJ [71]. The percentage of cells with cytoplasmic staining of phospho-S6 was manually counted.

### Statistical analyses

Statistical calculations were performed using GraphPad Prism version 6.07. Independent samples *t*-tests were used to compare results between two groups. Additional details are provided in Figure legends.

## SUPPLEMENTARY MATERIALS FIGURES AND TABLE


