# Supplementary material for: Efficacy of the combination of MEK and CDK4/6 inhibitors in vitro and in vivo in KRAS mutant colorectal cancer models
Source: Oncotarget. 2016 May 4;7(26):39595–608. doi: 10.18632/oncotarget.9153 (PMC5129956; doi:10.18632/oncotarget.9153)
Supplement: Supplementary file 1 [file oncotarget-07-39595-s001.pdf]

Efficacy of the combination of MEK and CDK4/6 inhibitors *in vitro* and *in vivo* in *KRAS* mutant colorectal cancer models

Supplementary Materials

Supplementary Table S1: Description of cell lines and doses of drugs used in colony formation assay

Cell line	*KRAS* Mut	*PIK3CA* Mut	Dose of palbociclib used for colony formation assay	Dose of MEK162 used for colony formation assay
HCT116	G13D	H1047R	400 nM	200 nM
Lovo	G13D	WT	200 nM	100 nM
SW480	G12V	WT	200 nM	100 nM
SKCO1	G12V	WT	100 nM	50 nM
SW403	G12V	WT	50 nM	25 nM
LS174T	G12D	H1047R	50 nM	25 nM
LS1034	A146T	WT	400 nM	200 nM
SW1116	G12A	WT	100 nM	50 nM
SW837	G12C	WT	400 nM	200 nM
SW948	Q61L	E542K	400 nM	200 nM
T84	G13D	E542K	400 nM	200 nM

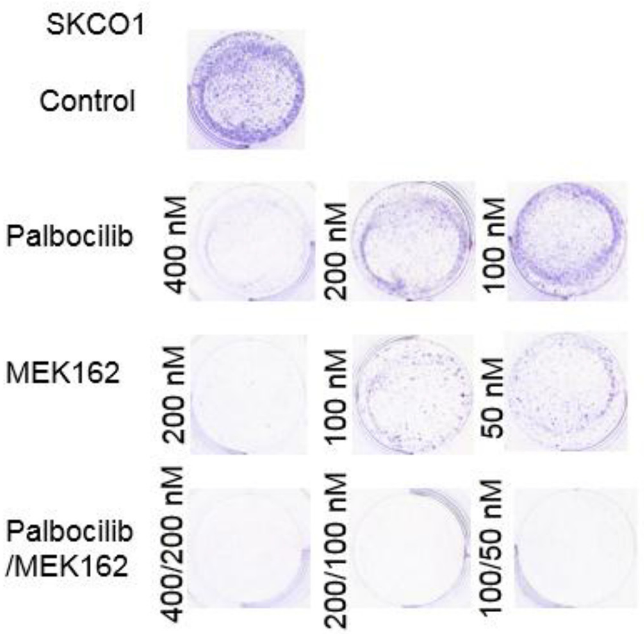

Supplementary Figure S1: Example of dose-finding experiment for clonogenic assays. The default dose selected for these experiments was 400 nM palbociclib, 200 nM MEK162, and the combination of the above. However, the effect of lower concentrations of palbociclib and MEK162 was also assessed in a preliminary dose-finding experiment, and a lower dose was selected for individual cell lines for further study in the clonogenic assays if a lower dose more clearly demonstrated efficacy of the combination, as demonstrated below for the SKCO1 cell line (in which the 100 nM palbociclib and 50 nM MEK162 dose was selected).

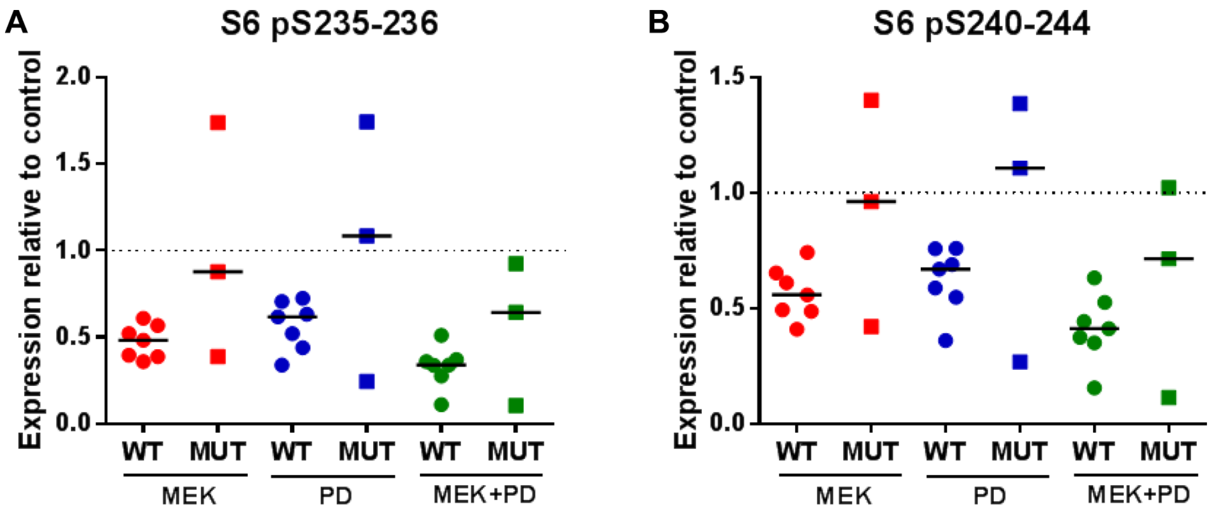

Supplementary Figure S2: Comparison of phospho-S6 levels as determined on RPPA between *PIK3CA* wild-type (WT, n = 7) and mutant (MUT, n = 3) cell lines. Cells were treated with DMSO control, MEK162 400 nM (MEK), palbociclib 200 nM (PD), or combination MEK162 400 nM and palbociclib 200 nM (MEK+PD) for 24 hours before protein lysates were collected. Values were normalized relative to DMSO-treated control. Scatterplots show median values of (**A**) phospho-serine235/236 or (**B**) phospho-serine240/244 as determined on RPPA. All comparisons of *PIK3CA* WT vs. MUT within each treatment were not statistically significant by Mann-Whitney U test.

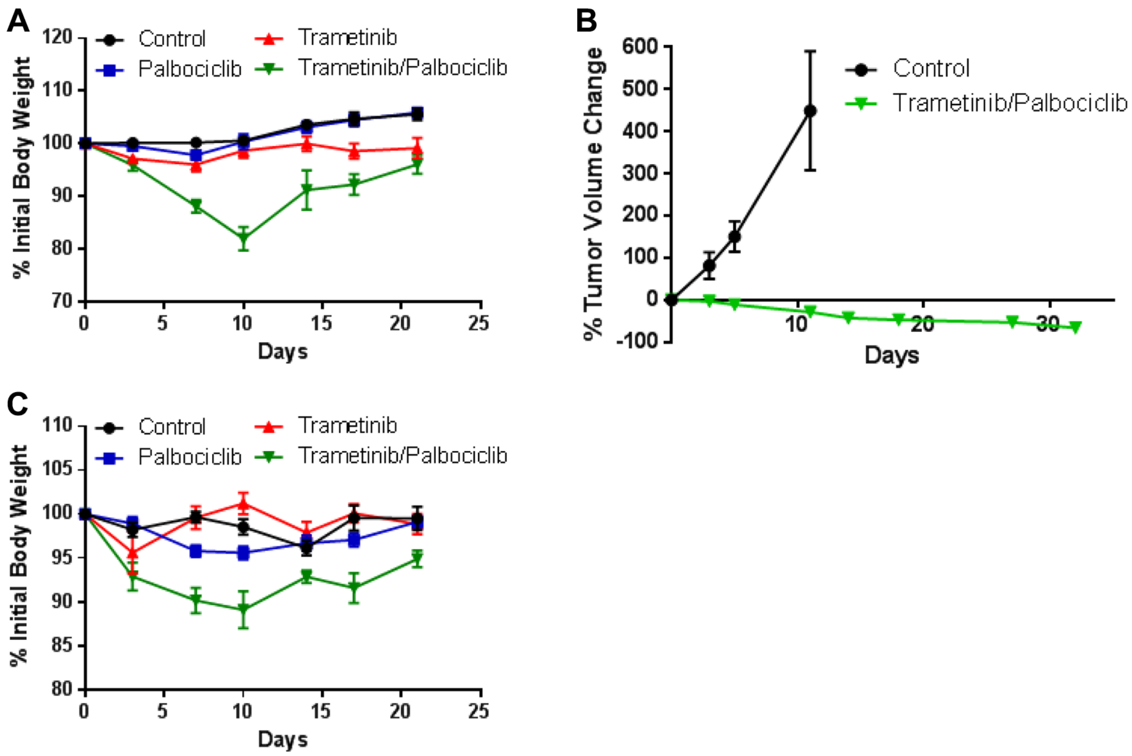

Supplementary Figure S3: (A) Body weight in KRAS-mutant Lovo cell line xenografts treated by oral gavage with vehicle control, trametinib 3 mg/kg every other day, palbociclib 100 mg/kg daily, or combination trametinib and palbociclib at same dosing regimens. $P < 0.002$ for combination vs. control and combination vs. palbociclib on days 7–21 by Student's t-test. $p < 0.03$ for combination vs. trametinib on days 7–17. (**B**) Lovo cell line xenografts were treated with control chow or chow containing trametinib + palbociclib, and tumor volume was measured longitudinally. (**C**) Body weight in KRAS-mutant PDXs treated by oral gavage with vehicle control, trametinib 3 mg/kg every other day, palbociclib 100 mg/kg daily, or combination trametinib and palbociclib at same dosing regimens. $P < 0.02$ for combination vs. control, combination vs. trametinib, or combination vs. palbociclib on days 7–21 by Student's t–test.

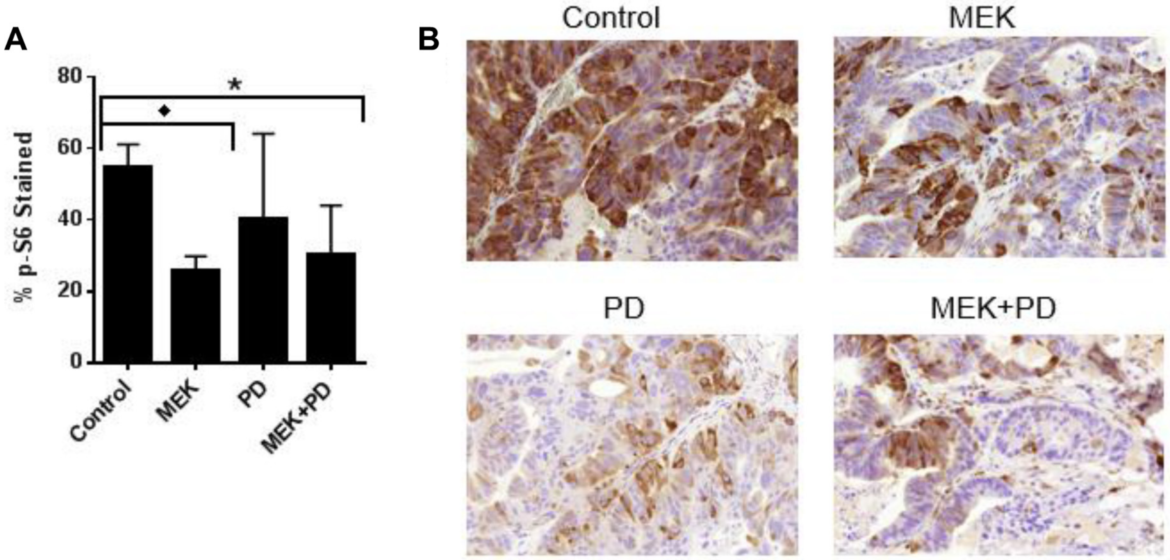

Supplementary Figure S4: (A) Percentage of cells stained by phospho-S6 antibody. The data represent mean value ± SD of samples from 3–4 mice. *$p \leq 0.02$, ♦$p < 0.003$ by Student's *t*-test. (**B**) Representative immunohistochemistry images for phospho-S6 staining.
